# Responses of Soil Collembolans to Land Degradation in a Black Soil Region in China

**DOI:** 10.3390/ijerph20064820

**Published:** 2023-03-09

**Authors:** Chen Ma, Runze Nie, Guoming Du

**Affiliations:** School of Public Administration and Law, Northeast Agricultural University, Harbin 150030, China; mac580@neau.edu.cn (C.M.);

**Keywords:** black soil, community characteristics, indicator, land degradation, soil Collembolans

## Abstract

Land degradation in black soil regions has a significant effect on belowground systems, and Collembolans can adequately indicate environmental changes in the soil. However, there is currently a knowledge gap in the literature regarding the responses of soil Collembolans to land degradation. In order to better understand this issue, in this study, a total of 180 soil Collembolan samples were collected from four habitats with varying degrees of land degradation in the Songnen Plain, namely a no land-degradation habitat (NLD), light land-degradation habitat (LLD), moderate land-degradation habitat (MLD) and severe land-degradation habitat (SLD). The results reveal that the different degrees of land degradation caused some differences in the taxonomic composition of the Collembolans; however, the majority of the Collembolan species are distributed relatively evenly. *Proisotoma minima* are always a dominant species during the study period. Seasonal variations are observed in the abundance, richness and diversity levels. In the severe land-degradation habitats (SLD), the abundance, richness, diversity and community complexity of the Collembolans are aways at the lowest levels. In addition, *Proisotoma minima* is negatively correlated with a majority of the species of Collembolans in the low levels of the land-degradation habitats, whereas they are positively correlated with most of the other species in the high levels. Epedaphic and euedaphic Collembolans responded to land degradation more obviously. The structural equation model (SEM) displays that soil Collembolan communities respond negatively to land degradation. Overall, our results provide implications that soil Collembolan communities are affected by land degradation, and that different taxa of soil Collembolans respond to degradation in numerous ways.

## 1. Introduction

Land degradation, a global issue affecting billions of people, is a phenomenon caused by a combination of natural forces and artificial activities [1]. Erosion by wind or water, salination, deforestation, high farming intensity and the overuse of pesticides and fertilizers are the main causes of land degradation [2]. Land degradation has many effects on the planet, with such effects felt all over the world. The main effects of land degradation can be soil erosion, salinization, acidification, and alkalization of the land, and finally desertification, and these can cause the lack of farmable land and the decline in land productivity [3]. Therefore, it is necessary to provide some insights into the mechanisms underlying degenerated soil, in order to prevent or reverse the effects of land degradation.

Land degradation can negatively impact the physical, chemical and biological quality of soil, with these effects today considered pervasive and systemic [4]. Generally, a visible effect of land degradation is decreases in soil thickness due to accelerated erosion [5]. With the thinning of soil, the repercussions of land degradation for soil environments start systematically from the soil’s physical properties. A previous study reveal that negative influences may have been observed in the formation and stability of soil aggregates along land-degradation gradients, and that the soil structure may have deteriorated [6]. After this, generally, soil nutrient stocks are changed and nutrient depletion is observed, and thus the amount, distribution and turnover rate of soil may be substantially influenced. Previous studies have demonstrated that land degradation may lead to a decline in soil C stocks and a high loss of soil N, as well as aggravating P deficiency [7,8,9]. These soil environmental changes can significantly affect the ecological processes and ecosystem multifunctionality of belowground systems, and thus soil organisms are also impacted. In this context, some previous studies have found that land degradation may significantly decrease the diversity of soil microbial communities [10,11]. Soil fauna is a critical component of soil organisms and has an irreplaceable role in the soil food web [12]. Nevertheless, a relatively small number of studies have investigated the effects of land degradation on soil fauna.

Collembolans (springtails), which inhabit soil and litter, belong to the three lineages of modern hexapods, and are the key consumers and decomposers in the terrestrial ecosystem [13]. Having a large population and a wide variety of species, they are one of the main components of soil fauna [14]. As they have different feeding guilds, Collembolans are found in various ecological niches, and these can directly cause them to act on ecological processes and ecosystem multifunctionality, thus playing crucial roles in belowground systems [15,16]. At the same time, their miniscule body size, fragile body strength and poor ability to migrate make Collembolans sensitive to changes in soil environments [17]. Once environmental factors lead to new microchanges, some significant responses are found in the taxonomic composition, community structure, diversity characteristics and distribution patterns of Collembolans. For instance, the restoration of vegetation can increase the abundance of soil Collembolans and cause their taxonomic composition to vary according to the different stages of vegetation restoration [18]. A previous study found a systematic shift in the composition traits of Collembolan communities across different land uses [19]. Consequently, Collembolans can adequately indicate environmental changes in belowground systems. It is an indisputable fact that land degradation can change the environment of belowground systems, thereby significantly altering soil properties. This inevitably affects soil Collembolan communities. However, there is currently a knowledge gap in the literature regarding the responses of soil Collembolans to land degradation.

Black soil, or mollisol in the USDA soil taxonomy, is characterized by its dark color and rich texture [20]. Due to its abundance of organic matter and nutrient elements, this type of soil is essential for the agricultural industry and has a variety of uses. Black soil can be found in many parts of the world, mainly the Central United States, Ukraine, Northeast China and Argentina. Songnen Plain, a typical black soil area, is one of the most important grain-producing regions in China. Recently, land degradation has been rapidly increasing in the Songnen Plain because of its flood-prone landform, high-intensity agricultural activities and non-conservation tillage system [21]. As a result, its soil environment is getting considerably worse. In light of this background, in order to better understand the responses of soil Collembolans to land degradation in a black soil region, we selected the Songnen Plain as the experimental site. Soil Collembolans were collected from four degrees of land-degradation habitats. We hypothesized that (H1) the changes in the soil Collembolan communities would be consistent with the land degradation in the black soil region, and (H2) that the soil Collembolan responses to the land degradation would differ across each of their taxa.

## 2. Materials and Methods

### 2.1. Site Description

In this study, the investigation was conducted in the northern part of the Songnen Plain (47°17′ N–47°54′ N, 125°92′ E–126°10′ E), where a great deal of black soil is widely distributed. This region is situated in the Xiao Hinggan Mountains and Songnen Plain intersection, and low hills account for a large proportion of its area. It is characterized by a continental monsoon climate, with long cold winters and short cool summers, and has a mean annual average temperature of 1.2 °C and mean annual precipitation of 490 mm. The soil type is mollisol, which is neutral or slightly acidic, with rich humus. Maize is most widely cultivated in the non-conservation tillage system used in the region, and thus land degradation is very common in the area. 

### 2.2. Sampling Design

To evaluate the responses of soil Collembolans to land degradation in a black soil region, habitats with different degrees of land degradation were selected among maize planting fields. Land degradation was divided into four degrees following the classification method of Sun et al. [22]. The habitats consisted of a no land-degradation habitat (NLD), light land-degradation habitat (LLD), moderate land-degradation habitat (MLD) and severe land-degradation habitat (SLD). Soil thickness was measured in the field, and the yield was monitored after harvest. The ecological parameters of each are shown in Table 1.

In this study, we collected samples in the spring (May), summer (July) and autumn (September) of 2021. Three independent stands (300 m^2^) and five replicate sample plots (1 m^2^) were randomly established. Soil core samples (25 × 25 × 20 cm) were taken in order to collect Collembolans in each plot. A total of 180 soil core samples (4 degrees × 3 replicated stands × 5 replicated plots × 3 sampling periods) were obtained. At the same time, soil samples were collected using soil augers in each plot.

### 2.3. Soil Collembolan Extraction and Identification

Soil core samples were extracted over 96 h via Tullgren funnel extractors. All of the Collembolan samples were preserved in 75% alcohol. After being cleared using Nesbitt’s fluid and mounted using Hoyer’s medium, soil Collembolan samples were made into slide specimens. Collembolans were counted using a Nikon SMZ745T stereoscopic microscope (Nikon, Tokyo, Japan) and a Nikon E200 biomicroscope (Nikon, Tokyo, Japan), and Collembolan specimens were identified at a species level [23,24,25].

### 2.4. Soil Analysis

After litters, roots and gravels were removed from the soil samples, each sample was air-dried and stored at room temperature. Soil properties were measured using conventional standards [26]. Briefly, K_2_Cr_2_O_7_–H_2_SO_4_ digestion and FeSO_4_ titration were applied to determine soil organic matter (SOM); the total nitrogen (TN) and available phosphorus (AP) values of the soil were measured using a SEAL AA3 auto analyzer (SEAL, Mequon, WI, USA); the pipette method was applied to measure contents of soil clay particles and sand grains.

### 2.5. Statistical Analysis

A Venn diagram was created to illustrate the differences in soil Collembolan taxonomic composition within the four land-degradation degrees, with endemic species being manually screened and a diagram being created using the “VennDiagram” R package [27]. To analyze the effects of land-degradation degrees on the soil Collembolan community complexity, a network analysis was performed based on Spearman’s rank correlation matrix, using Cytoscape 3.9.1 [28].

To compare the differences in the soil Collembolan abundance (individuals per m^−2^) caused by land degradation, the Tukey’s HSD test was conducted using the “car” R package [29]. To test the effects of land-degradation degrees on soil Collembolan richness and diversity, rarefaction curves were created using EstimateS 9.1 [30]. A heatmap of the hierarchical clustering was created to evaluate the distribution patterns of soil Collembolan species among the four land-degradation habitats, using the “pheatmap” R package [31]. The hierarchical clustering in the heatmap was based on the unweighted pair-group method with a mathematical mean algorithm (UPGMA), and the cluster analysis was conducted using the “stats” R package [32].

In addition, Pearson’s correlations were calculated to reveal the relationships between each soil Collembolan genus and different environmental variables via the “corrplot” R package [33]. In order to determine direct and indirect interaction effects between independent and measured variables in a single model, structural equation models (SEMs) were employed using the “lavaan” R package [34].

## 3. Results

### 3.1. Taxonomic Composition and Community Structure

During the study period, 10,341 individual soil Collembolans were collected from the four land-degradation habitats: these belonged to 30 species, 26 genera, 12 families and three orders ( Supplementary Material Table S1). In total, the dominant species were found to be *Proisotoma minima* (30.20%) and *Isotomiella minor* (13.46%), and the common species included *Parisotoma dichaeta* (8.89%), *Proisotoma* sp.1 (8.89%), *Ceratophysella* sp.1 (5.81%), *Folsomia candida* (5.42%), *Bionychiurus* sp.1 (5.24%), *Protaphorura* sp.1 (4.18%), *Micronychiurus* sp.1 (3.89%), *Homidia quadrimaculata* (3.47%), *Xenylla* sp.1 (1.91%), *Metaphorura affinis* (1.26%) and *Tomocerus kinoshitai* (1.12%). Additionally, the residual 17 species were rare species, accounting for 6.28% of the total number of individuals.

**Figure 1:**
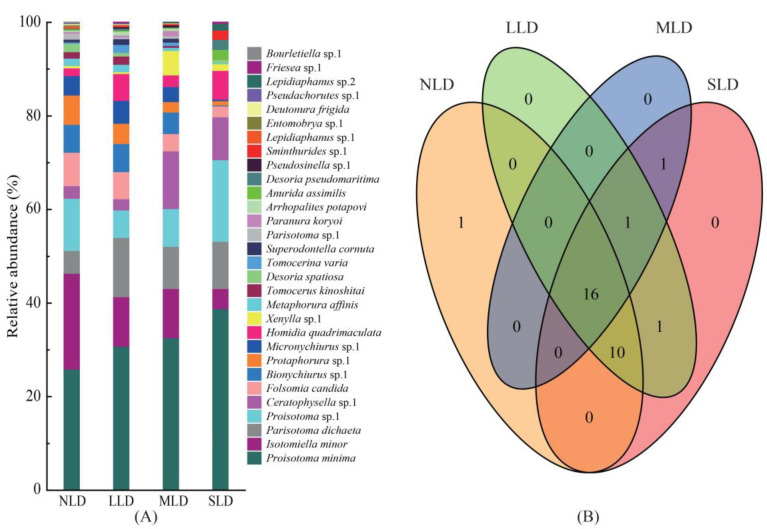
Characteristics of soil Collembolan communities. (**A**) The relative abundance was defined as the percentage of soil Collembolan communities in different degrees of land degradation; (**B**) Venn diagram of the number of shared and unique soil Collembolan species in different degrees of land degradation. The numbers in the circles indicate either the unique number of species in a habitat or the number of shared taxa in the overlapping regions. Soil Collembolans were counted form three seasons during the study period. NLD, no land-degradation habitat; LLD, light land-degradation habitat; MLD, moderate land-degradation habitat; SLD, severe land-degradation habitat.

At the species level, there were both similar dominant species and different taxonomic compositions among the four land-degradation habitats (Figure 1A). *Proisotoma minima* were always a dominant species during the study period. *Isotomiella minor* were observed to be a dominant species in the no land-degradation habitat (NLD), light land-degradation habitat (LLD) and moderate land-degradation habitat (MLD), whereas they were a common species (4.32%) in the severe land-degradation habitat (SLD). *Parisotoma dichaeta* were only a dominant species in the LLD (12.64%) and SLD (10.07%). At the same time, *Proisotoma* sp.1 were only a dominant species in the NLD (11.15%) and SLD (17.45%). *Ceratophysella* sp.1 was a dominant species (12.34%) only in the MLD.

Across the different land-degradation habitats, both unique and shared species of soil Collembolans were found, as illustrated in the Venn diagram shown in Figure 1B. All four habitats had 16 species (*Proisotoma minima*, *Isotomiella minor*, *Parisotoma dichaeta*, *Proisotoma* sp.1, *Ceratophysella* sp.1, *Folsomia candida*, *Bionychiurus* sp.1, *Protaphorura* sp.1, *Micronychiurus* sp.1, *Homidia quadrimaculata*, *Xenylla* sp.1, *Metaphorura affinis*, *Desoria spatiosa*, *Anurida assimilis*, *Desoria pseudomaritima* and *Sminthurides* sp.1) in common. *Pseudosinella* sp.1 was only collected in the LLD and MLD, while *Friesea* sp.1 was only found in the LLD and SLD. *Bourletiella* sp.1 was the only unique species found in the NLD.

**Figure 2:**
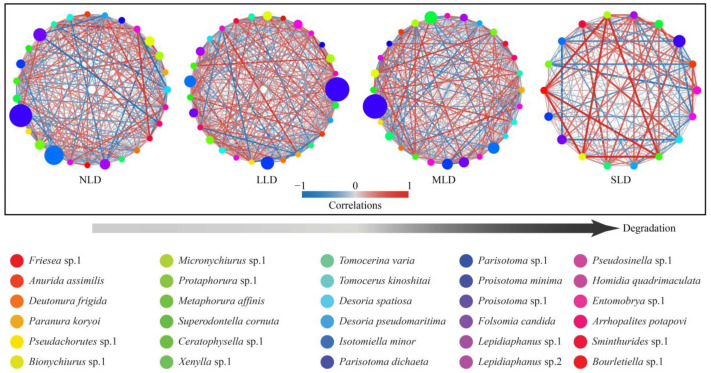
Network visualization of soil Collembolan community structure. The size of the circles represents the abundance of soil Collembolans, and the different colors represent different species. Soil Collembolans were counted form three seasons during the study period. NLD, no land-degradation habitat; LLD, light land-degradation habitat; MLD, moderate land-degradation habitat; SLD, severe land-degradation habitat.

The community structure and key topological features of the soil Collembolans were summarized using network analysis (Figure 2). It was found that community complexity declined as the degree of land degradation increased; among the habitats, this was most obvious in the SLD. At the same time, we found that various interspecific relationships existed between each habitat. For instance, *Proisotoma minima* was negatively correlated with a majority of the species of soil Collembolans found in the habitats with low levels of land degradation (NLD and LLD), yet it was positively correlated with most of the other species in the habitats with high levels of land degradation (MLD and SLD).

### 3.2. Diversity Characteristics and Distribution Patterns

During the study period, 337,300 m^−2^ (27 species) Collembolan specimens were collected in the NLD; 333,200 m^−2^ (29 species) in the LLD; 308,000 m^−2^ (28 species) in the MLD; and 55,600 m^−2^ (18 species) in the SLD. Among these habitats, the NLD had the most abundance, and the LLD displayed the most richness. In contrast, the SLD had the least abundance and richness.

**Figure 3:**
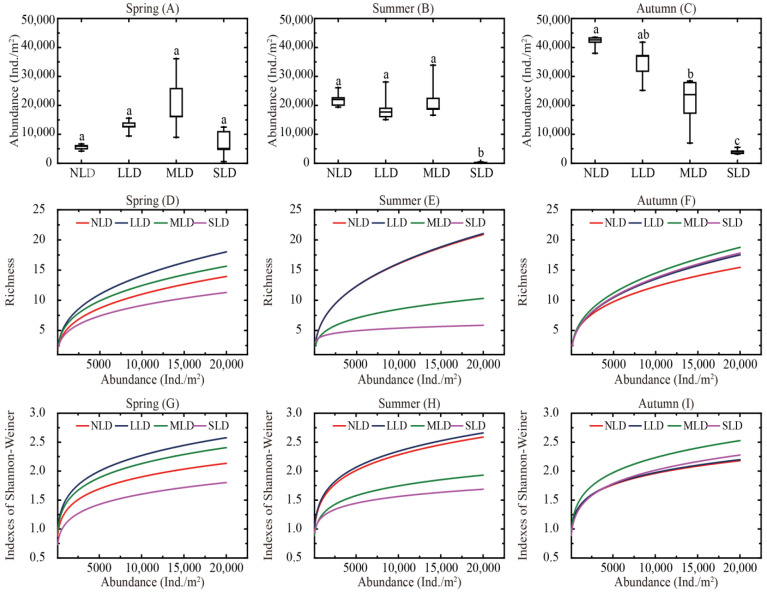
Diversity characteristics of soil Collembolans during the study period. (**A**–**C**) Box and whisker plots illustrating the medians (line in the box), 25th and 75th percentiles (box), 10th and 90th percentiles (outer lines), and mean values (dots) of abundance (**A**) of soil Collembolans in different land-degradation habitats. (**D**–**I**) Rarefaction curves of soil Collembolan richness and Shannon–Wiener index assemblage in different land-degradation habitats. Same letter(s) indicate no significantly different between each land-degradation habitats at *p* < 0.05 by Tukey’s HSD. NLD, no land-degradation habitat; LLD, light land-degradation habitat; MLD, moderate land-degradation habitat; SLD, severe land-degradation habitat.

During the different seasons, the diversity characteristics of the soil Collembolans differed across the four habitats (Figure 3). The abundance data of the soil Collembolans are shown in Figure 3A–C. The abundance of the Collembolans from the SLD was consistently at the lowest levels during the study period, with these levels being significant in summer and autumn (*p* < 0.05). During spring, significant differences were not found across all the four habitats (*p* > 0.05), and the highest abundance was observed in the MLD. In summer, there were no significant differences among the NLD, LLD and MLD (*p* > 0.05), and the MLD again had the greatest abundance. During autumn, the abundance of the NLD was the highest, and was significantly greater than the abundance of the MLD and SLD (*p* < 0.05).

The rarefaction curves of the soil Collembolan richness and diversity are shown in Figure 3D–I. All the curves approached plateaus, and this indicated that the majority of the soil Collembolan species were collected in these areas. The richness and diversity of the soil Collembolans varied across the different land-degradation habitats. In spring and summer, the richness and diversity of the SLD were both obviously lower than the richness and diversity of other habitats, whereas distinctions in the richness and diversity of the habitats were not clear during autumn.

**Figure 4:**
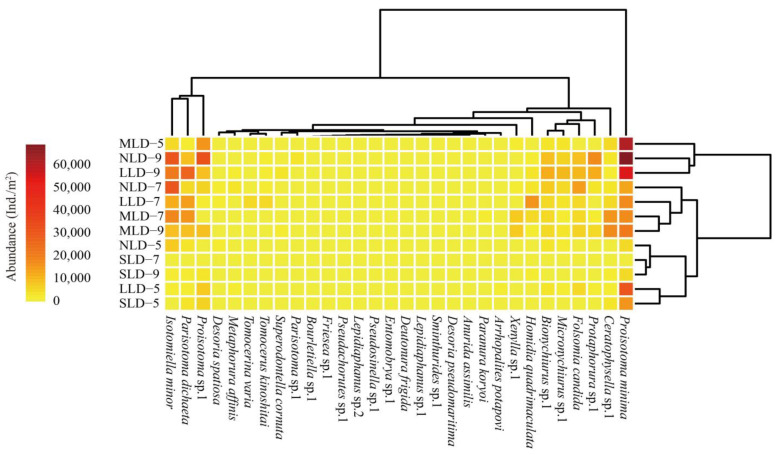
Abundance heatmap of log(x + 1)-normalized soil Collembolans in different land-degradation habitats. Dendrogram of sample sites based on similarity along right axis; dendrogram of soil Collembolan species based on similarity along upper axis. Colors represent abundance of soil Collembolans. Five indicate spring; seven indicate summer; nine indicate autumn. NLD, no land-degradation habitat; LLD, light land-degradation habitat; MLD, moderate land-degradation habitat; SLD, severe land-degradation habitat.

The heat map shown in Figure 4 illustrates the distribution patterns of the soil Collembolans from the four land-degradation habitats during the study period. These four land degradation habitats were divisible into three clusters, specifically as follows: major similarities existed among the spring MLD, autumn NLD and LLD; most of the habitats (except the SLD) in summer and the MLD in autumn were divisible into a cluster; the residue sites were similar to each other. At the same time, the soil Collembolan communities were also divisible into three clusters. *Proisotoma minima* was only able to make up one cluster. *Isotomiella minor*, *Proisotoma* sp.1 and *Parisotoma dichaeta* formed another cluster. Finally, other species were divisible into a third cluster, and this indicated that the majority of Collembolan species were distributed relatively evenly. 

### 3.3. Correlations of Soil Collembolans with Land Degradation

**Figure 5:**
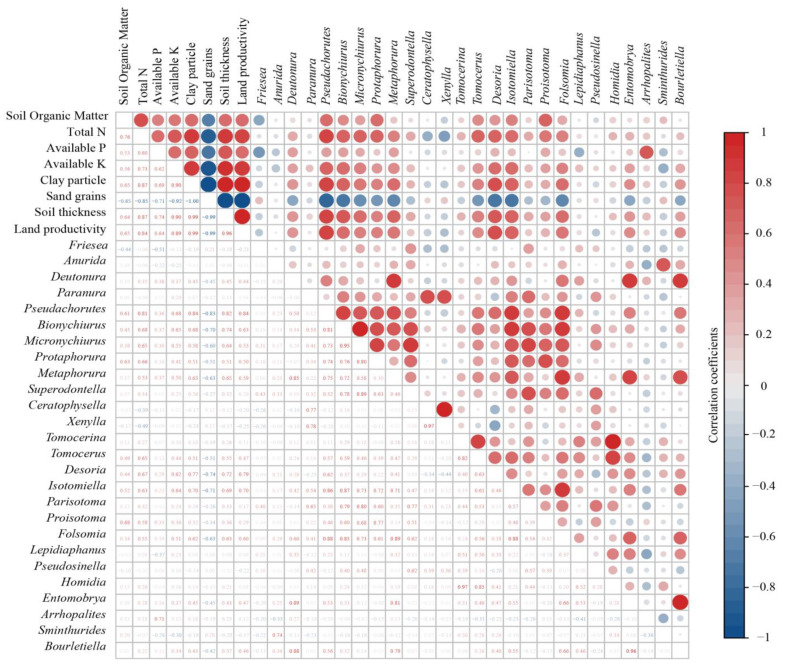
Pearson’s correlations among soil organic matter, total N, available P, available K, clay particles, sand grains, soil thickness, land productivity and soil Collembolans at the genus level. The size of the circles located in the upper half of figure represents the correlation coefficients. The numbers located in the lower half of figure are the correlation coefficients. A correlation coefficient less than 0.53241 indicates a significant correlation (*p* < 0.05).

As illustrated by Pearson’s correlations, different environmental variables were correlated with each genus of soil Collembolans in numerous ways (Figure 5). *Pseudachorutes*, *Bionychiurus*, *Micronychiurus*, *Protaphorura*, *Metaphorura*, *Tomocerus*, *Desoria* and *Isotomiella* were significantly correlated with the majority of the soil environmental variables caused by land degradation (*p* < 0.05). This revealed that these genera might exhibit relatively sensitive responses to land degradation. At the same time, we found that most of the genera were significantly negatively correlated with the contents of soil sand grains (*p* < 0.05). This indicated that land sandification might be a major limiting factor for soil Collembolans. In addition, soil available P was found not to be correlated with most of the genera, except *Arrhopalites*, which had a significantly positive correlation with soil available P (*p* < 0.05).

**Figure 6:**
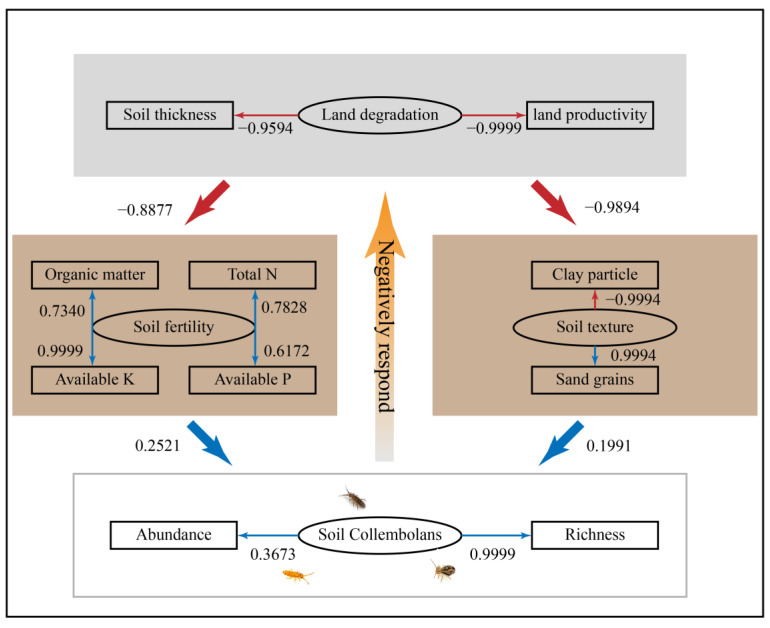
Structural equation model relating land degradation, soil fertility, soil texture and soil Collembolan communities (χ^2^/*df* = 2.145, *p* = 0.365, GFI = 0.907, CFI = 0.919 and RMSEA = 0.017). The rectangles represent the observed variables (soil organic matter, total N, available P, available K, clay particles, sand grains, soil thickness, land productivity (yield of maize), abundance and richness); the ovals represent latent variables (land degradation, soil fertility, soil texture and soil Collembolan communities). The red arrows indicate a negative correlation between each variable; the blue arrows indicate a positive correlation between each variable. The numbers indicate regression weights.

A structural equation model (SEM) was designed to evaluate the correlations among the land degradation (soil thickness and land productivity), soil fertility (soil organic matter, total N, available P and available K), soil texture (clay particles and sand grains) and soil Collembolan communities (abundance and richness) (Figure 6). The final model provided an excellent fit (χ^2^/df = 2.145, *p* = 0.365, GFI = 0.907, CFI = 0.919 and RMSEA = 0.017). The SEM found that land degradation significantly negatively affected the soil fertility (regression weight = −0.8877) and soil texture (regression weight = −0.8877). At the same time, the soil Collembolan communities were positively influenced by the soil fertility (regression weight = 0.2521) and soil texture (regression weight = 0.1991). Therefore, this model indicated that land degradation may have impacted the soil Collembolan communities by affecting the soil fertility and soil texture.

## 4. Discussion

### 4.1. Effects of Land Degradation on Soil Collembolan Communities

In this study, we observed that the soil Collembolan specimens from the severe land-degradation habitat (SLD), whether regarding their abundance, richness, diversity or community complexity, were always at the lowest levels compared to the other land-degradation habitats (NLD, LLD and MLD). Further, the community complexity found among the different habitats was consistent with the land degradation. This indicated that land degradation may have limited the soil Collembolan communities in the black soil study region. These findings were in line with our hypothesis that changes in soil Collembolan communities would be consistent with the land degradation present in the black soil study region (H1), and agreed with the previous study of soil bacterial communities [35]. Previous studies revealed that land surface organic matter could provide abundant food resources for Collembolans, and that it was the key factor that directly affects soil Collembolan communities [36]. In this study, we observed that the land degradation led to visible declines in the contents of soil organic matter (Table 1). Consequently, the Collembolan living environment was short on food resources, which resulted in a deficiency of the Collembolans in the SLD. Additionally, a previous study revealed that Collembolans partially breathe through their skin, and thus need a moist environment [37]. In the SLD, we found that the proportion of sand grains was greater than that of other habitats, which might have rapidly increased the soil water runoff and created relatively drier conditions. As a result, the soil Collembolan communities were further restricted in this severe land-degradation habitat. 

Further, in this study, 27 species of Collembolans were collected in the NLD, 29 species in the LLD and 28 species in the MLD. These findings were partially out of line with our first hypothesis (H1). The “intermediate disturbance hypothesis” predicts that maximum levels of biodiversity should be observed under some intermediate disturbance frequency in ecological communities, which permits more species invasions and colonization [38]. Compared with the other land degradation habitats, the LLD habitat was at the intermediate disturbance level, which may have promoted the prosperity of the species of soil Collembolans. At the same time, the Venn diagram and heat map constructed in the study illustrated that the majority of the Collembolans were distributed relatively evenly, indicating that land degradation did not cause the obvious change in fauna composition observed in the study region (Figure 1B and Figure 4). Previous studies demonstrated that the fauna composition of soil Collembolans was dependent on environmental conditions on the regional scale [39]. Since all the sites included in this study were located in the Songnen Plain, they had the same environmental conditions. Consequently, similarities across the soil Collembolans were observed.

### 4.2. Responses of Soil Collembolans to Land Degradation

We found that different soil environmental variables were correlated with each genus of soil Collembolans in numerous ways, and this confirmed our hypothesis that responses to land degradation would differ across the soil Collembolan taxa (H2). Previous studies revealed that life histories, feeding guilds, propagation characteristics and adaptability mechanisms differed dramatically across soil Collembolan taxa [40,41,42]. Consequently, in this study, a variety of responses to land degradation were observed across the different genera of soil Collembolans. At the same time, we found that *Pseudachorutes*, *Bionychiurus*, *Micronychiurus*, *Protaphorura*, *Metaphorura*, *Tomocerus*, *Desoria* and *Isotomiella* were significantly correlated with the majority of the soil environmental variables caused by land degradation (*p* < 0.05) (Figure 5). Soil Collembolans could be categorized into three life forms: euedaphic (subsurface active); hemiedaphic (within the soil and partly on the surface) and epedaphic (surface-active) [43,44,45]. In this study, *Bionychiurus*, *Micronychiurus*, *Protaphorura* and *Metaphorura* are examples of euedaphic Collembolans, which prefer to live in deep soils. *Tomocerus*, *Desoria* and *Isotomiella* are examples of epedaphic Collembolans, which prefer to live on the soil surface. Meanwhile, hemiedaphic Collembolans, which were intermediate dwellers, exhibit strong endurance to environmental changes. Consequently, in this study, the euedaphic and epedaphic Collembolans exhibited relatively sensitive responses to land degradation. In addition, *Bourletiella* sp.1 was the only unique species found in the NLD. *Bourletiella*, which belongs to the order Symphypleona, are typical herbivorous and epedaphic Collembolans [46]. Herbivorous Collembolans take fresh organic matter as their main food. Abundant soil nutrients may produce plenty of fresh organic matter, and thus habitats with low levels of land degradation may be beneficial to Symphypleona.

In this study, we determined that *Proisotoma minima*, a dominant species in all the habitats, was negatively correlated with the majority of soil Collembolan species in the habitats with low levels of land degradation (NLD and LLD), yet they were positively correlated with most of the other species in the habitats with high levels of land degradation (MLD and SLD) (Figure 2). *Proisotoma*, primary decomposers that feed on litter and some fungi, had an abundant population, and were thus located in a relatively low niche [46]. In the habitats with low levels of land degradation, the environmental conditions were relatively better than that of other habitats; consequently, a large number of *Proisotoma minima* were able to occupy the living spaces of species with identical niches. As a result, they were negatively correlated with other species of soil Collembolans. In the habitats with high levels of land degradation, the environmental conditions were relatively worse, which might have released some living space for Collembolans with identical niches, and thus this might have led to the positive correlation found between *Proisotoma minima* and other species. Consequently, the relationship between *Proisotoma minima* and other Collembolans indicated that land degradation was present in the black soil study region.

We found that soil Collembolan communities might have responded negatively to land degradation, because the land degradation changed the soil fertility and soil texture (Figure 6). Land degradation could cause a great deal of soil clay to leach, and it radically reduces the ability to preserve soil and water [47,48]. As a result, considerable organic matter left belowground ecosystems as root exudates and litter, which led to sequential declines in soil fertility [49]. Soil fertility is vitally important in supplying sufficient food for Collembolans, while loam is crucial in providing relatively wetter conditions at the same time [50,51]. Consequently, we found that the soil Collembolans responded negatively to land degradation in the study area.

In addition, this study still has a lot of room for improvement in the responses of soil Collembolan to land degradation. The morphological characteristics of soil Collembolan have been confirmed to diversify with environmental factors, and thus these characteristics are functional [52]. Consequently, functional traits associated with changes in soil Collembolan communities may indicate the occurrence of land degradation. However, there is a relatively small number of studies available at present. Therefore, in the future, it is necessary to strengthen the studies on the functional traits of soil Collembolans responding to land degradation, and finally put forward a deeper explanation of the relationships between soil Collembolans and environmental changes.

## 5. Conclusions

In summary, in this study, it was found that the soil Collembolan communities may have exhibited negative responses to land degradation in the study region. The Collembolan specimens’ taxonomic composition varied across the different land-degradation habitats, which were located in a black soil region of the Songnen Plain, but the majority of the Collembolan species were distributed relatively evenly across this region. The abundance, richness, diversity and community complexity of the soil Collembolans from the severe land-degradation habitat were always at the lowest levels compared to the other habitats. Further, seasonal variations were observed in the abundance, richness and diversity levels of the soil Collembolans. *Proisotoma minima* was negatively correlated with a majority of the species of soil Collembolans in the habitats with low levels of land degradation, whereas they were positively correlated with most of the other species in the habitats with high levels of land degradation. Additionally, each genus of soil Collembolans responded to the land degradation in numerous ways, with the epedaphic and euedaphic Collembolans responding more obviously to it. The findings of this study have implications for the study of the relationship between land degradation and soil Collembolans, and can provide some assistance in developing biodiversity guidelines for farmland protection in black soil regions.

## Figures and Tables

**Table 1:** The ecological parameters in different land-degradation habitats.

	SOM (g/kg)	TN (g/kg)	AP (mg/kg)	AK (mg/kg)	CP (%)	SG (%)	Soil Thickness (cm)	Yield (t/hm^2^)
NLD	40.67	6.90	13.43	274.01	37.76	59.63	101.05	1.25
LLD	33.11	6.43	13.24	215.32	25.96	71.12	85.88	1.00
MLD	30.01	3.40	11.10	208.62	15.33	80.37	62.57	0.85
SLD	23.57	2.96	6.34	134.91	7.23	90.55	40.33	0.75

Note: SOM, soil organic matter; TN, total N; AP, available P; AK, available K; CP, clay particles; SG, sand grains; Yield, yield of maize. NLD, no land-degradation habitat; LLD, light land-degradation habitat; MLD, moderate land-degradation habitat; SLD, severe land-degradation habitat.

## Data Availability

Not applicable.

## Supplementary Materials

The following supporting information can be downloaded at: https://www.mdpi.com/article/10.3390/ijerph20064820/s1, Table S1: Taxonomic composition of Collembolans in different land degradation habitats.
