# Supplementary material for: Responses of Soil Collembolans to Land Degradation in a Black Soil Region in China
Source: Int J Environ Res Public Health. 2023 Mar 9;20(6):4820. doi: 10.3390/ijerph20064820 (PMC10048822; doi:10.3390/ijerph20064820)
Supplement: Supplementary file 1 [file ijerph-20-04820-s001.zip › ijerph-2255688-supplementary.pdf]

Supplementary material Table S1 Taxonomic Composition of Collembolan in different land degradation habitats

Taxonomy of Collembolan				Abundance （Ind./m^2）			
Oders	Families	Genus	Species	NLD	LLD	MLD	SLD
Poduromorpha	Neanuridae	*Anurida*	*Anurida assimilis*	1000	700	600	1200
		Deutonura	*Deutonura frigida*	700	500	200	0
		Friesea	*Friesea* sp.1	0	100	0	100
		Paranura	*Paranura koryoi*	1300	1200	3300	0
		Pseudachorutes	*Pseudachorutes* sp.1	700	400	100	0
	Onychiuridae	*Bionychiurus*	*Bionychiurus* sp.1	20000	20000	14100	100
		Micronychiurus	*Micronychiurus* sp.1	14000	16200	9800	200
		Protaphorura	*Protaphorura* sp.1	21200	14500	7000	500
	Tullbergiidae	*Metaphorura*	*Metaphorura affinis*	5500	5400	2000	100
	Odontellidae	*Superodontella*	*Superodontella cornuta*	1900	4000	2500	0
	Hypogastruridae	*Ceratophysella*	*Ceratophysella* sp.1	9000	8000	38000	5100
		Xenylla	*Xenylla* sp.1	1600	1200	16100	800
Entomobryomorpha	Tomoceridae	*Tomocerus*	*Tomocerus kinoshitai*	4500	5900	1200	0
		Tomocerina	*Tomocerina varia*	1100	5700	2200	0
	Isotomidae	*Desoria*	*Desoria spatiosa*	6100	2600	100	400
			Desoria pseudomaritima	500	1400	200	1200
		Isotomiella	*Isotomiella minor*	69100	35300	32400	2400
		Parisotoma	*Parisotoma dichaeta*	16400	42100	27800	5600
			Parisotoma sp.1	4200	1300	1900	0
		Proisotoma	*Proisotoma minima*	87500	102700	100500	21600
			Proisotoma sp.1	37600	19700	24900	9700
		Folsomia	*Folsomia candida*	24200	19200	11300	1300
	Lepidocyrtidae	*Lepidiaphanus*	*Lepidiaphanus* sp.1	1000	600	700	0
			Lepidiaphanus sp.2	0	300	100	800
		Pseudosinella	*Pseudosinella* sp.1	0	1000	1500	0

	Entomobryidae	*Homidia*	*Homidia quadrimaculata*	5600	19200	7700	3400
		Entomobrya	*Entomobrya* sp.1	1100	400	100	0
	Arropalitidae	*Arrhopalites*	*Arrhopalites potapovi*	900	2900	1400	0
Symphypleona	Bourletiellidae	*Bourletiella*	*Bourletiella* sp.1	200	0	0	0
	Sminthurididae	*Sminthurides*	*Sminthurides* sp.1	400	700	300	1100

Note：NLD, no land degradation habitat; LLD, light land degradation habitat; MLD, moderate land degradation habitat; SLD, severe land degradation habitat.
